# SPECT/CT image‐based dosimetry for Yttrium‐90 radionuclide therapy: Application to treatment response

**DOI:** 10.1002/acm2.12400

**Published:** 2018-07-01

**Authors:** Peter S. Potrebko, Ravi Shridhar, Matthew C. Biagioli, William F. Sensakovic, George Andl, Jan Poleszczuk, Timothy H. Fox

**Affiliations:** ^1^ College of Medicine University of Central Florida Orlando FL USA; ^2^ Department of Physics University of Central Florida Orlando FL USA; ^3^ Department of Radiation Oncology Florida Hospital Orlando FL USA; ^4^ Department of Radiology Florida Hospital Orlando FL USA; ^5^ Varian Medical Systems Atlanta GA USA; ^6^ Nalecz Institute of Biocybernetics and Biomedical Engineering Polish Academy of Sciences Warsaw Poland

**Keywords:** Yttrium‐90, dosimetry, SPECT

## Abstract

This work demonstrates the efficacy of voxel‐based ^90^Y microsphere dosimetry utilizing post‐therapy SPECT/CT imaging and applies it to the prediction of treatment response for the management of patients with hepatocellular carcinoma (HCC). A ^90^Y microsphere dosimetry navigator (RapidSphere) within a commercial platform (Velocity, Varian Medical Systems) was demonstrated for three microsphere cases that were imaged using optimized bremsstrahlung SPECT/CT. For each case, the ^90^Y SPECT/CT was registered to follow‐up diagnostic MR/CT using deformable image registration. The voxel‐based dose distribution was computed using the local deposition method with known injected activity. The system allowed the visualization of the isodose distributions on any of the registered image datasets and the calculation of dose‐volume histograms (DVHs). The dosimetric analysis illustrated high local doses that are characteristic of blood‐flow directed brachytherapy. In the first case, the HCC mass demonstrated a complete response to treatment indicated by a necrotic region in follow‐up MR imaging. This result was dosimetrically predicted since the gross tumor volume (GTV) was well covered by the prescription isodose volume (V150 Gy = 85%). The second case illustrated a partial response to treatment which was characterized by incomplete necrosis of an HCC mass and a remaining area of solid enhancement in follow‐up MR imaging. This result was predicted by dosimetric analysis because the GTV demonstrated incomplete coverage by the prescription isodose volume (V470 Gy = 18%). The third case demonstrated extrahepatic activity. The dosimetry indicated that the prescription (125 Gy) isodose region extended outside of the liver into the duodenum (178 Gy maximum dose). This was predictive of toxicity as the patient later developed a duodenal ulcer. The ability to predict outcomes and complications using deformable image registration, calculated isodose distributions, and DVHs, points to the clinical utility of patient‐specific dose calculations for ^90^Y radioembolization treatment planning.

## 1 INTRODUCTION

Radionuclide therapy using Yttrium‐90 (^90^Y) microspheres has emerged as an effective treatment modality for the management of patients with primary and metastatic hepatocellular carcinoma (HCC).^1^ SIR‐Spheres is FDA‐PMA (Food and Drug Administration‐Premarket Approval) approved for metastatic colorectal cancer to the liver.^2^ TheraSphere is FDA approved under HDE (humanitarian device exemption) for radiation treatment or as neo‐adjuvant to surgery or transplantation in patients with HCC who can have appropriately placed hepatic arterial catheters.^3^ This device is indicated for HCC patients with partial or branch portal vein thrombosis/occlusion when clinical evaluation warrants the treatment.^4^

Current practice uses manufacturer‐recommended prescription activity and dose calculations, derived from the Medical Internal Radiation Dose (MIRD) Committee of the Society of Nuclear Medicine, which are based on the size of the intended treatment volume.^5^ Despite positive results from published studies,^6^, ^7^ the reported doses assume that the activity distribution within the treatment volume is uniform. Quantitative patient‐specific ^90^Y dosimetry has been studied using bremsstrahlung single photon emission computed tomography (SPECT)/computed tomography (CT) 8, 9, 10, 11 and positron emission tomography (PET)/CT.^12^, ^13^, ^14^, ^15^, ^16^ However, such dosimetric studies have utilized research software that is not readily available to the medical community and have not illustrated scenarios where voxel‐based dosimetry could be used to predict treatment response.

In this study, we demonstrate how commercial voxel‐based absorbed dose calculation software applied to post‐therapy ^90^Y bremsstrahlung SPECT imaging can facilitate dosimetric verification during the course of ^90^Y therapy. This work is meant as a proof‐of‐concept to highlight the potential clinical merits of using a patient‐specific ^90^Y dose calculation to quantify the treatment plan. Specifically, we demonstrate how voxel‐based dosimetry can be used to predict treatment response and treatment complications. To our knowledge, this is the first time a study has illustrated the use of isodose distributions and dose‐volume histograms in ^90^Y therapy to predict not only tumor response but also normal tissue complications.

## 2 METHODS

**Figure 1:**
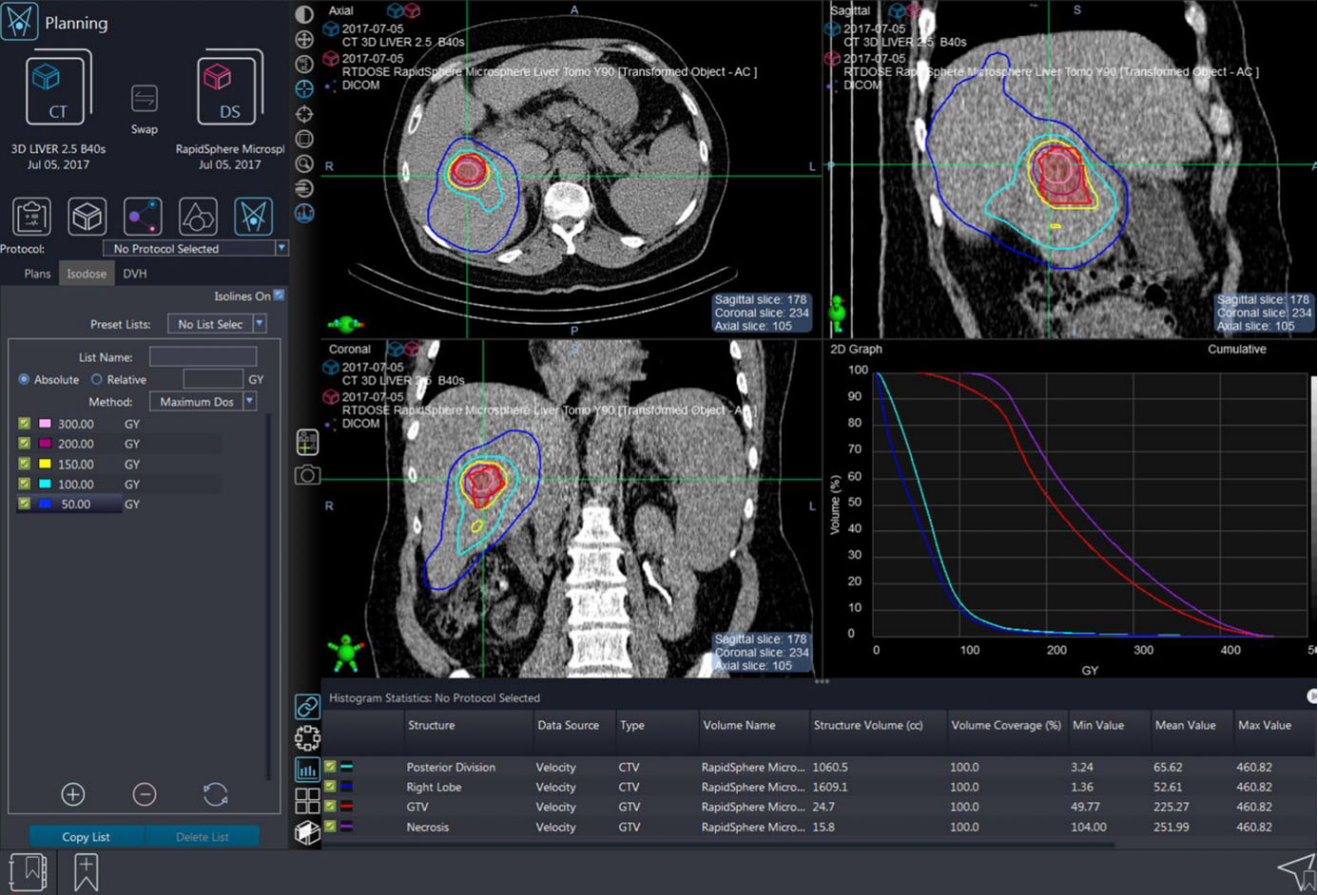
The commercial ^90^Y microsphere dosimetry navigator (RapidSphere) allows for rigid or deformable image registration, SPECT/CT‐based voxel dosimetry, and visualization of isodoses and dose‐volume histograms.

Three cases were selected to demonstrate the utility of patient‐specific dosimetry in ^90^Y therapy. All patients underwent imaging and clinical evaluation before treatment. This included arterial mapping and abdominal Technetium‐99m‐macroaggregated albumin (^99m^Tc‐MAA) SPECT/CT to assess the potential for extrahepatic uptake and determine the feasibility, safety, and number of injections required for treatment. Planar SPECT imaging was used for the liver–lung shunt calculation. For therapy, ^90^Y glass microspheres (TheraSphere, BTG Inc., Ottawa, Canada) were administered with an activity calculated according to the manufacturer's specifications.^3^ Within 90 min after the administration of the ^90^Y, a bremsstrahlung SPECT/CT was acquired using a Siemens Symbia T6 (Siemens Medical Solutions Inc., Malvern, PA) with an energy window of 90–125 keV based on a highly optimized acquisition and reconstruction protocol published by Siman et al.^17^ The SPECT projection images were acquired with a matrix size of 128 × 128, 4.8 mm pixel size, 28 s/view for 128 views over 360°. The SPECT images were reconstructed using the manufacturer's three‐dimensional ordered subset expectation maximization algorithm (Flash3D, Siemens) using 8 iterations with 16 subsets, geometric collimator response modeling, CT‐based attenuation correction using effective energy of the imaging window, dual‐energy window‐based scatter correction, and a 9.6 mm FWHM postreconstruction Gaussian filter.^17^ Dosimetry was performed using the ^90^Y microsphere dosimetry navigator (RapidSphere) within Velocity (Varian Medical Systems, Atlanta, GA) as illustrated in Fig. 1.

The first step within Velocity was to deformably register the ^90^Y SPECT/CT images to the pre‐ and post‐treatment diagnostic images (MRI and CT). The deformable registration algorithm applies multiresolution free‐form deformations based on cubic spline interpolation between sparse, uniformly distributed control points as its transformation model.^18^, ^19^ The registration provided a one‐to‐one correlation between voxels on different images and time points, allowing for mapping of anatomical data and structure sets from diagnostic to follow‐up imaging. Velocity automatically segmented the patient's external body contour on the SPECT/CT which was later used to cumulate the SPECT counts within the patient (excluding the lungs) for the dose calculation as described below.

The second step used the ^90^Y dosimetry navigator to calculate the absorbed dose distribution from the ^90^Y SPECT/CT images. The ^90^Y dose calculation used the local deposition method (LDM),^20^, ^21^ which was previously demonstrated to be the most accurate when using SPECT imaging.^22^ In this technique, ^90^Y β‐particles released by decay within a voxel deposit all energy locally with the same voxel. This is an accurate approximation considering that the mean range of β‐particles in tissue is 3.8 mm which is within the typical SPECT voxel size (4.8 mm cubic voxels in our study). The absorbed dose within a voxel is

(1) $$D_{\mathrm{voxel}}=\frac{A(1-LSF)T_{1/2}E_{\mathrm{avg}}C_{\mathrm{voxel}}}{\Delta V\rho \ln\left(2\right)C_{\mathrm{total}}}$$

where *A *= injected activity, *LSF* = lung shunt fraction, *T*_1/2_ = ^90^Y physical half‐life (64.24 hours), *E*_*avg*_ **= average β‐particle energy per disintegration (0.935 MeV), *C*_*voxel*_ **= SPECT counts within voxel, Δ*V *= voxel volume, ρ = tissue density, and *C*_*total*_ **= total SPECT counts within the patient (excluding the lungs). The derivation of Eq. (1) can be found in the Appendix. The only patient‐specific parameters that had to be manually entered into the software prior to the dose calculation were the injected activity (GBq) and the lung shunt fraction (expressed as %). The default tissue density was set to that of soft tissue (1.04 g/cm^3^). However, the software does provide the ability to enter tissue‐specific densities for different contoured structures.

The last step in the ^90^Y dosimetry navigator was to evaluate the isodose distributions superimposed on the deformably registered image datasets and generate dose‐volume histograms (DVHs) for the contoured structures. It is important to note that the isodose distribution was not truncated within the liver contour, which allowed for the evaluation of extrahepatic dose.

## 3 RESULTS

### 3.A Case #1 — Complete Response

**Figure 2:**
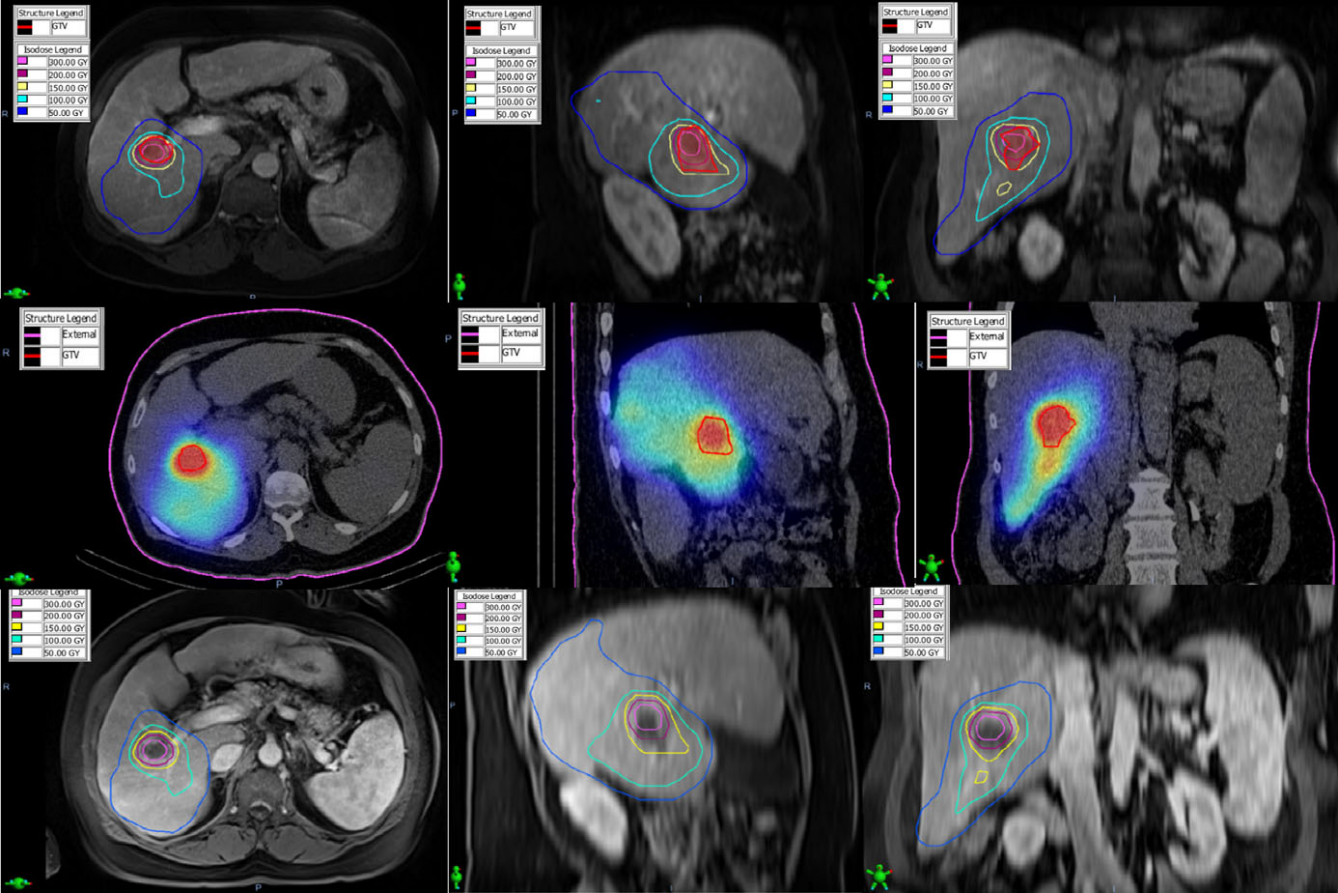
The deformably registered pre‐treatment diagnostic MR (top), post‐treatment SPECT/CT (middle), and the 5‐week follow‐up MR (bottom) for Case #1. Notice the GTV (red outline) is well covered by the prescription (150 Gy) isodose volume in the pretreatment MR and post‐treatment SPECT/CT which corresponds to a necrotic region in the follow‐up MR.

**Figure 3:**
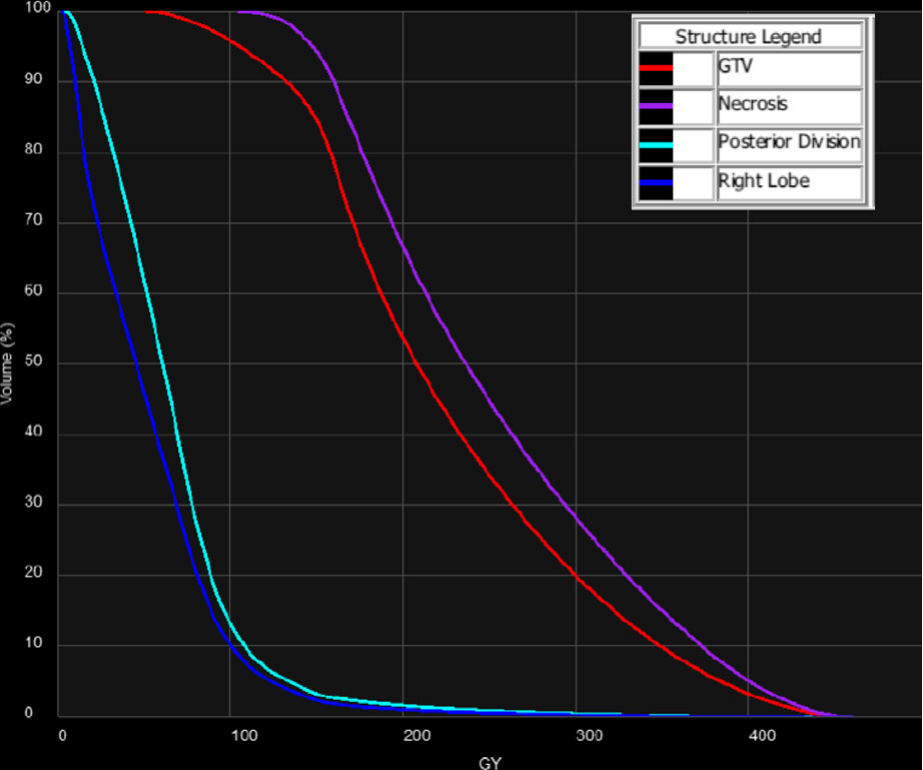
Dose‐volume histograms of the GTV, necrotic region, posterior division, and right lobe for Case #1.

The patient was a 64‐year‐old male with hepatitis C induced cirrhosis and HCC. This patient was determined to be unresectable due to the degree of cirrhosis and was referred for radionuclide therapy using ^90^Y microspheres as a bridge to transplant. The patient underwent an angiographic workup and SPECT/CT‐based lung shunt study to satisfy the criteria for treatment. The lung shunt fraction was determined to be 3.9%. An HCC gross target volume (GTV) in the right posterior division (segments 6/7) was contoured on the contrast‐enhanced T1‐weighted MR. A dose of 150 Gy was prescribed to the right posterior division (1000 cc volume). The patient received 3.217 GBq of ^90^Y using 2.8 million microspheres. Fig. 2 illustrates the deformably registered pretreatment diagnostic MR, post‐treatment SPECT/CT, and the 5‐week follow‐up MR with the superimposed ^90^Y isodose distribution. Notice that the ≥150 Gy dose region provided good coverage (V150 Gy = 85%) of the contoured GTV in the pretreatment MR and post‐treatment SPECT/CT which is also apparent from the DVH in Fig. 3. The 5‐week follow‐up MR demonstrated complete response to treatment characterized by a necrotic region where the mass was originally located. This necrotic region was relatively well covered (V150 Gy = 94%) by the ≥150 Gy dose region. The DVH of the contoured necrotic region is shown in Fig. 3.

### 3.B Case #2 — Partial response

**Figure 4:**
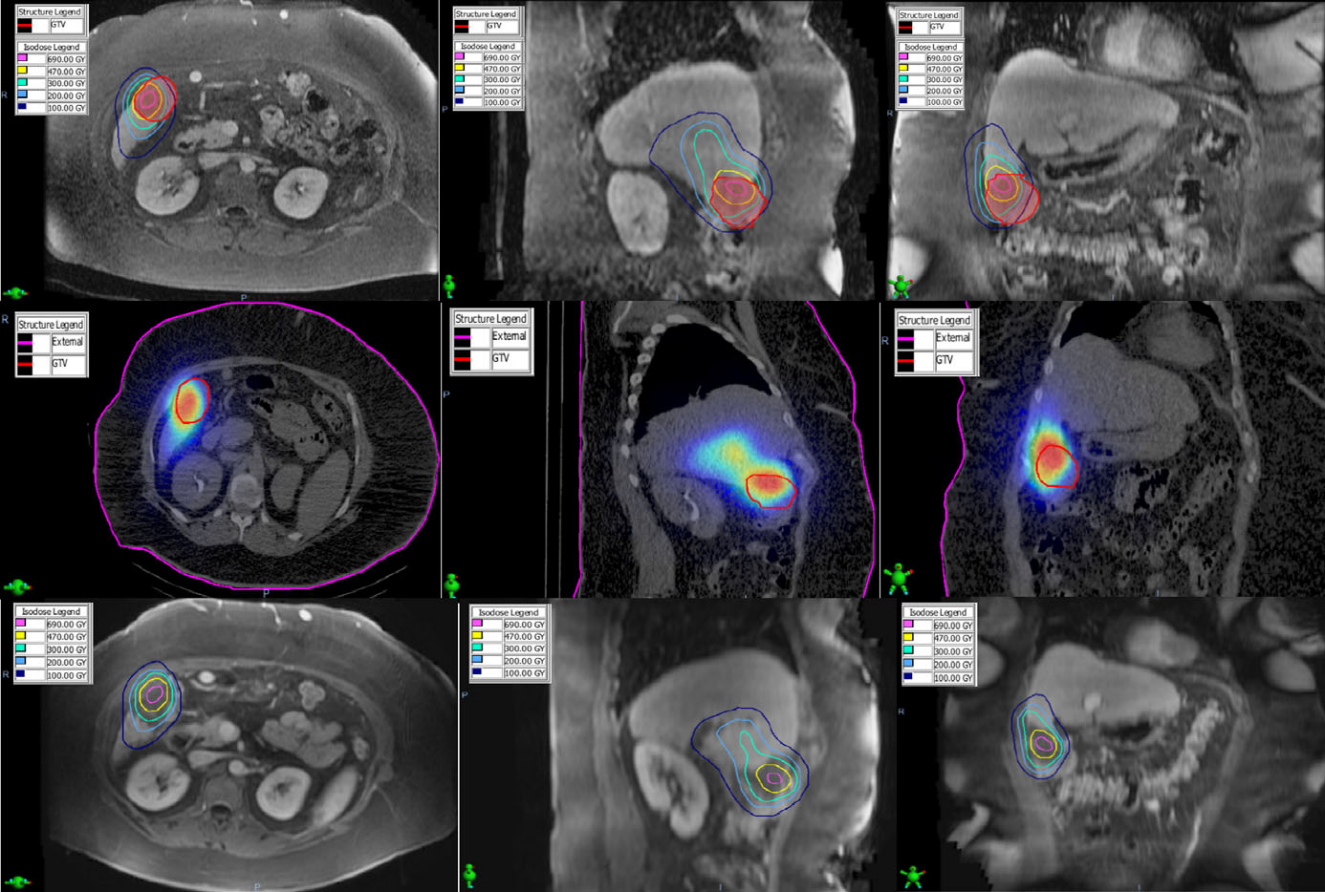
The deformably registered pretreatment diagnostic MR (top), post‐treatment SPECT/CT (middle), and the 6‐week follow‐up MR (bottom) for Case #2. Notice incomplete coverage of the GTV (red outline) by the prescription (470 Gy) isodose volume in the pretreatment MR and post‐treatment SPECT/CT which corresponds to a partially necrotic region with residual enhancing tumor in the follow‐up MR.

**Figure 5:**
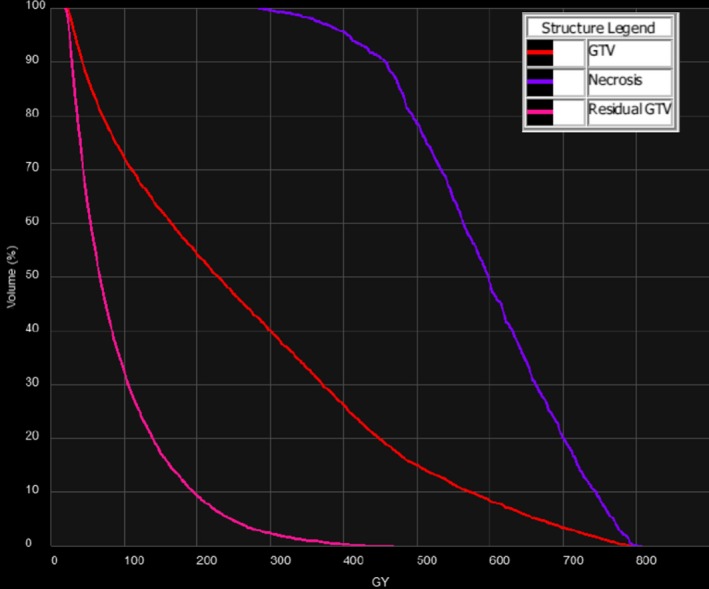
Dose‐volume histograms of the GTV, necrotic region, and residual GTV for Case #2.

The patient was a 60‐year‐old female with HCC. A SPECT/CT‐based lung shunt study was performed. The lung shunt fraction was determined to be 3.3%. An HCC gross target volume (GTV) invading both segments 5/6 was noted on the diagnostic MR. A dose of 690 Gy and 470 Gy was prescribed to segment 5 (160 cc volume) and segment 6 (230 cc volume), respectively. The patient received 2.258 GBq of ^90^Y to segment 5 and 2.223 GBq of ^90^Y to segment 6 using 1.2 million microspheres each. Fig. 4 illustrates the deformably registered pretreatment diagnostic MR, post‐treatment SPECT/CT, and the 6‐week follow‐up MR with the superimposed ^90^Y isodose distribution. Notice that the ≥470 Gy dose region provided incomplete coverage (V470 Gy = 18%) of the contoured GTV in the pretreatment MR and post‐treatment SPECT/CT which is also apparent from the DVH in Fig. 5. The possibility of incomplete coverage was suspected at the time of arterial mapping due to the presence of omental vessels supplying the tumor that could not be catheterized. The 6‐week follow‐up MR demonstrated that the GTV had a partial response to treatment. The mass was incompletely necrotic and there were still areas of solid enhancement. The ≥470 Gy dose region did not cover the contoured residual tumor (V470 Gy = 0%), however, the necrotic region was well covered (V470 Gy = 87%) which is apparent in the DVHs in Fig. 5.

### 3.C Case #3 — Extrahepatic activity

**Figure 6:**
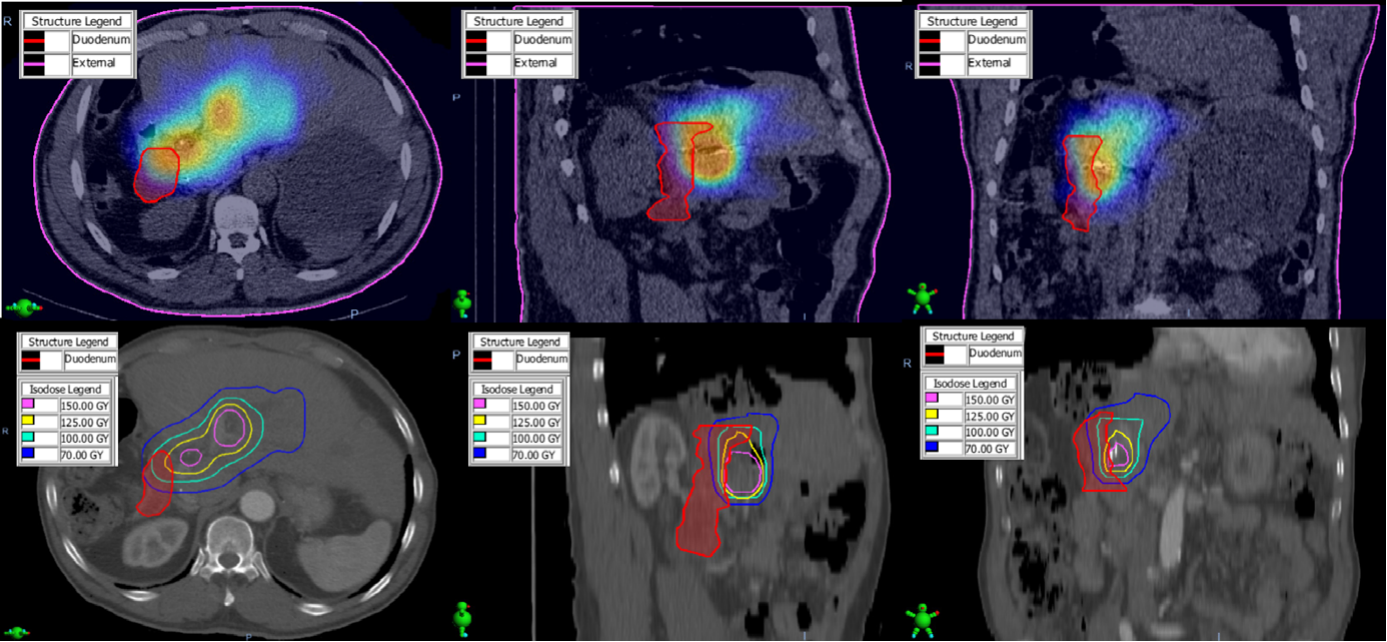
The deformably registered post‐treatment SPECT/CT (top), and the 11‐week follow‐up CT (bottom) with the superimposed ^90^Y isodose distribution for Case #3. Notice the prescription isodose volume (125 Gy) extends outside the liver into the duodenum.

**Figure 7:**
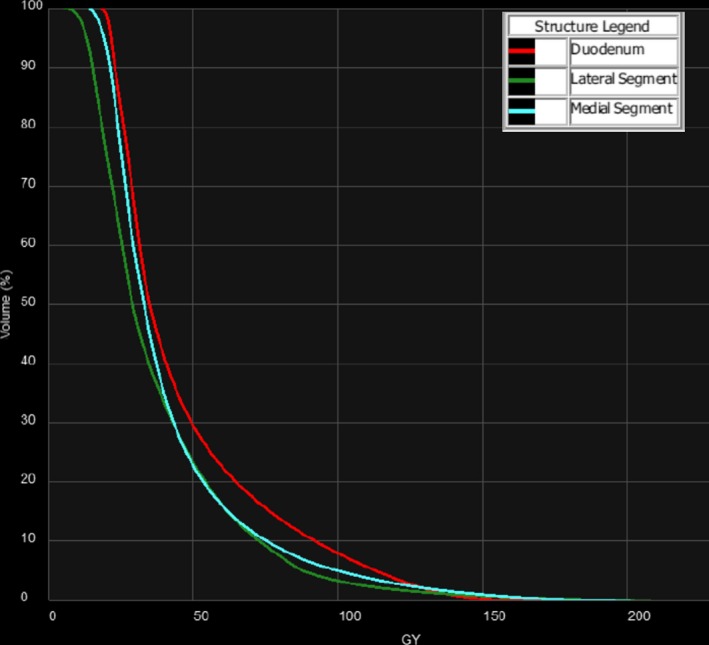
Dose‐volume histograms of the duodenum, and medial and lateral segments for Case #3.

The patient was a 69‐year‐old male with HCC. The lung shunt fraction was determined to be 3.7% from the SPECT/CT study. A dose of 125 Gy was prescribed to the medial and lateral segments (1400 cc volume). The patient received 3.563 GBq of ^90^Y using 3.2 million microspheres. Fig. 6 illustrates the deformably registered post‐treatment SPECT/CT and the 11‐week follow‐up CT with the superimposed ^90^Y isodose distribution. Extrahepatic activity was noted after treatment when new supraduodenal arteries had formed since the arterial mapping study and there was a catheter malfunction at the time of injection. Notice that the ≥125 Gy dose region extended outside the liver into the duodenum. The DVH of the duodenum, with a maximum dose of 178 Gy, is shown in Fig. 7. This patient developed a duodenal ulcer because of this high radiation dose.

## 4 DISCUSSION

Radiation Oncology treatment modalities require an accurate estimation of the dose in Gray delivered to tumors and normal tissue. This fundamental principle also applies to ^90^Y therapy which is a form of brachytherapy. However, ^90^Y radioembolization has been practiced without a strong understanding of the actual quantity of radiation exposure in liver tumors, healthy liver tissue, and tissue surrounding the liver. It is our hope that this work will be a catalyst to opening a new era of patient‐specific ^90^Y dosimetry using commercially available software.

There is some debate on whether to use bremsstrahlung SPECT/CT or PET/CT for ^90^Y postradioembolization imaging. Since β‐radiation emitted by ^90^Y interacts with body tissues resulting in Bremsstrahlung radiation, SPECT/CT has traditionally been regarded as the gold‐standard modality to image the biodistribution of this radionuclide. However, the low photon yield and continuous nature of the Bremsstrahlung x‐ray spectrum make SPECT imaging involving ^90^Y technically challenging.^17^ On the other hand, the ^90^Y decay to the 0^+^ excited state and then ground state of ^90^Zr may result in emission of a positron‐electron pair. Despite the branching ratio of pair‐production being quite small (∼32 ppm), more recent studies have shown ^90^Y PET/CT to be feasible in phantoms and patients.^23^ Advanced correction techniques for scatter, random, and attenuation effects that are clinically available for ^18^F PET can be directly applied to ^90^Y PET.^24^ Elschot et al. demonstrated that time‐of‐flight PET‐based dose estimates were more accurate than SPECT‐based dose estimates, which produced large underestimations in high‐dose regions.^15^ Conversely, Yue et al. concluded that SPECT/CT, with appropriate reconstruction methods, and PET/CT were comparable in terms of estimating total activity in the liver and activity distributions within treated volumes.^12^ However, these authors also found that PET overestimated the activity distribution in regions with low activity. This overestimation may be clinically insignificant when considering ^90^Y therapy alone, however, it may become important in situations where ^90^Y and external beam treatments are given subsequently because there is a need to more accurately quantify delivered doses to surrounding tissue. Collateral dose to normal liver from ^90^Y therapy is nontrivial and can have clinical implications.^13^

Case #2 demonstrated only partial response to ^90^Y radioembolization possibly due to the nonuniform distribution of microspheres. As a result, this patient will undergo external beam radiotherapy to treat the residual tumor. The microsphere dosimetry navigator can produce a composite ^90^Y and external beam dose distribution and has the unique ability to calculate extrahepatic ^90^Y dose using different tissue densities. This capability may be important for estimating ^90^Y dose within the lung and at the liver–lung interface 8 and is currently unavailable in other ^90^Y commercial dosimetry software. This topic will be the subject of a future investigation.

There are several limitations to our study. First, this study was designed to demonstrate proof‐of‐concept and illustrate clinical impact of a commercial ^90^Y dose calculation method. A large patient cohort study is needed to better quantify potential benefits of a post‐therapy dosimetric analysis and demonstrate a dose–response relationship for tumor and normal tissue using this software. Srinivas et al. demonstrated a possible dose–response trend for tumors based on a retrospective study of only 56 patients with HCC.^13^ However, the authors used research software which was not commercially available. It is our hope that the widespread availability of commercial microsphere dosimetry software will be the catalyst for larger studies. The impact of respiratory motion was not considered in our study. Bastiaannet et al. demonstrated that respiration is the predominant degrading effect on ^90^Y SPECT image quantification and can be alleviated through respiratory gating.^25^ A similar conclusion was presented by Siman et al. for ^90^Y PET imaging.^26^ Therefore, future patient cohort studies will need to consider respiratory motion in order to more accurately quantify the delivered dose from both SPECT and PET post‐therapy imaging.

## 5 CONCLUSION

In this study, we demonstrated that voxel‐based dosimetry for ^90^Y microsphere therapy allows for quantitative quality assurance of the delivered treatment. As with any other brachytherapy modality, dosimetric verification during the course of ^90^Y therapy is vital to help us better understand the source of treatment outcome and toxicity. Given the utility of ^90^Y dosimetry and the availability of commercial software packages for easy implementation, we believe ^90^Y dosimetry should become standard clinical practice.

## CONFLICT OF INTEREST

P. Potrebko is the recipient of a Varian Medical Systems grant. G. Andl and T. Fox are employed by Varian Medical Systems. R. Shridhar is a consultant for BTG International Ltd.

## APPENDIX 1

### 1.1

The following describes the derivation of Eq. (1). The absorbed dose within a voxel is

(A1) $$D_{\mathrm{voxel}}=\frac{E_{\mathrm{voxel}}}{m_{\mathrm{voxel}}}$$

where *E*_*voxel*_ = energy deposited within a voxel, *m*_*voxel*_ = mass of voxel

However,

(A2) $$m_{\mathrm{voxel}}=\rho \;\Delta V$$

where ρ = tissue density, Δ*V* = volume of voxel

Also,

(A3) $$E_{\mathrm{voxel}}=E_{\mathrm{avg}}N$$

where *E*_*avg*_ = average β‐particle energy per disintegration, *N *= total number of disintegrations from a given amount of activity as a function of time within the voxel, integrated over all time.

But,

(A4) $$N=\frac{A_{\mathrm{voxel}}}{\lambda }$$

where *A*_*voxel*_ = initial activity within the voxel, λ = decay constant

And,

(A5) $$\lambda =\frac{\ln(2)}{T_{1/2}}$$

where *T*_1/2_ = physical half‐life (physical decay only, that is, no biological clearance is assumed in the case of Y‐90 microspheres).

However,

(A6) $$A_{\mathrm{voxel}}=\frac{C_{\mathrm{voxel}}}{C_{\mathrm{Total}}}A_{\mathrm{Total}}$$

where *C*_*voxel*_ = SPECT counts within voxel, *C*_*Total*_ = total SPECT counts within patient (excluding lungs), *A*_*Total*_ = total activity within the patient (excluding lungs).

Finally,

(A7) $$A_{\mathrm{Total}}=A\left(1-LSF\right)$$

where *A *= injected activity, *LSF* = lung shunt fraction

Therefore, using Eqs. (A2), (A3), (A4), (A5), (A6), and (A7) into Eq. (A1) we obtain:

(A8) $$D_{\mathrm{voxel}}=\frac{A\left(1-LSF\right)T_{1/2}E_{\mathrm{avg}}C_{\mathrm{voxel}}}{\Delta V\rho \ln\left(2\right)C_{\mathrm{total}}}$$
